# Nigral Iron Deposition Influences Disease Severity by Modulating the Effect of Parkinson’s Disease on Brain Networks

**DOI:** 10.3233/JPD-223372

**Published:** 2022-12-16

**Authors:** Jiaqi Wen, Tao Guo, Jingjing Wu, Xueqin Bai, Cheng Zhou, Haoting Wu, Xiaocao Liu, Jingwen Chen, Zhengye Cao, Luyan Gu, Jiali Pu, Baorong Zhang, Minming Zhang, Xiaojun Guan, Xiaojun Xu

**Affiliations:** a Department of Radiology, The Second Affiliated Hospital, Zhejiang University School of Medicine, Hangzhou, China; b Department of Neurology, The Second Affiliated Hospital, Zhejiang University School of Medicine, Hangzhou, China

**Keywords:** Parkinson’s disease, nigral iron, brain networks, quantitative susceptibility mapping, functional magnetic resonance imaging, interaction

## Abstract

**Background:**

In Parkinson’s disease (PD), excessive iron deposition in the substantia nigra may exacerbate α-synuclein aggregation, facilitating the degeneration of dopaminergic neurons and their neural projection.

**Objective:**

To investigate the interaction effect between nigral iron deposition and PD status on brain networks.

**Methods:**

Eighty-five PD patients and 140 normal controls (NC) were included. Network function and nigral iron were measured using multi-modality magnetic resonance imaging. According to the median of nigral magnetic susceptibility of NC (0.095 ppm), PD and NC were respectively divided into high and low nigral iron group. The main and interaction effects were investigated by mixed effect analysis.

**Results:**

The main effect of disease was observed in basal ganglia network (BGN) and visual network (VN). The interaction effect between nigral iron and PD status was observed in left inferior frontal gyrus and left insular lobe in BGN, as well as right middle occipital gyrus, right superior temporal gyrus, and bilateral cuneus in VN. Furthermore, multiple mediation analysis revealed that the functional connectivity of interaction effect clusters in BGN and medial VN partially mediated the relationship between nigral iron and Unified Parkinson’s Disease Rating Scale II score.

**Conclusion:**

Our study demonstrates an interaction of nigral iron deposition and PD status on brain networks, that is, nigral iron deposition is associated with the change of brain network configuration exclusively when in PD. We identified a potential causal mediation pathway for iron to affect disease severity that was mediated by both BGN dysfunction and VN hyperfunction in PD.

## INTRODUCTION

Parkinson’s disease (PD) is one of the most common neurodegenerative diseases [1]. The death of dopaminergic neurons in the substantia nigra (SN) and aberrant aggregation of α-synuclein are the core pathological changes of PD [2]. A large number of histochemical studies support the hypothesis that the overload of nigral iron may be an underlying cause for the loss of dopaminergic neurons [3]. Pathologically, the nigral iron deposition could induce irreparable neurodegenerative processes through neurotoxic and oxidative reactions [4]. Since iron might modify α-synuclein *in vitro*, the deposition of iron in the SN may increase the aggregation of a-synuclein, thus aggravating its neurotoxicity [5]. It has been found that nigral iron deposition could indirectly affects the topological properties of the functional network in PD through the mediation role of dysfunctional functional connectivity (FC) between the striatum [6]. However, there has been little study about the interaction effect of PD status and nigral iron on brain functional network. In other words, we do not know whether iron deposition in the SN affects brain functional network differently in PD in comparison with that in normal elderly.

It is worth noting that, according to the well-established dopaminergic loop of the nigrostriatal pathway, basal ganglia receives projections from dopaminergic neurons in the SN, and then connects to the cerebral cortex, generating a typical basal ganglia network (BGN) [7], which is the most frequently disrupted brain network in PD [7]. Because brain function configuration is highly organized and is working as a large-scale network complex composed of invaluable brain networks, intensive regulations between BGN and other networks [8], including functional perturbation and complementary changes, would be expected to reserve the balance of a “normal” function configuration in PD. We speculated that, under PD status, the existence of excessive nigral iron deposition would additionally interrupt the constructed network balance and further change the brain function configuration, which might be associated with a poorer clinical profile.

To well address this hypothesis, multi-modality magnetic resonance imaging (MRI) with multiple specific brain information becomes a non-invasive candidate in the clinical investigation. We used resting-state functional magnetic resonance imaging (rsfMRI), which have been widely employed to simulate the human brain as a large-scale network complex, to capture brain spontaneous fluctuations of PD patients and the normal elderly [9], and further decomposed them into many independent functional networks using independent component analysis (ICA) [10], e.g., BGN [11]. Quantitative susceptibility mapping (QSM), as a highly repeatable MRI technique that measures the spatial distribution of tissue magnetic susceptibility [12], has been well established as the gold standard to quantifying brain iron *in vivo* [12]. By taking these advantages of MRI techniques, some mostly recent evidence showed the possibility to study the brain function in consideration of the influence of subcortical iron content [6, 13]. Therefore, we used QSM to measure iron content in the SN, and explore the potential effect of nigral iron deposition on brain function configuration under PD status.

This study aimed to decode the respective effect of PD status and nigral iron deposition and their potential interaction effect on brain functional networks using multi-modality MRI technology, and further explore their relationships with disease severity.

## MATERIALS AND METHODS

### Participants

All PD patients and normal controls (NC) signed informed consent forms in accordance with the approval of the Medical Ethic Committee of the Second Affiliated Hospital of Zhejiang University School of Medicine. The diagnosis of PD was made by an experienced neurologist (B.Z.) according to UK Parkinson’s Disease Society Brain Bank criteria [14].

One hundred and twenty-nine PD patients and 157 sex- and age-matched normal elderly recruited from August 2014 to August 2017 were included in this study. Participants with a history of neurologic or psychiatric disorders, brain trauma, or general exclusion criteria for MR scanning and analyzing were excluded from the study. Specifically, 44 PD and 17 NC were excluded for the following reasons: 1) with significant motion artifact during scanning, *n* = 3; 2) with severe brain atrophy or enlarged ventricles, *n* = 9; 3) with other neurologic or psychiatric disease history, *n* = 1; 4) with metal dentures, *n* = 14; 5) with incomplete fMRI data, *n* = 8; 6) with failed rsfMRI preprocessing, *n* = 17; 7) with T1-weighted image artifacts, *n* = 1; 8) with significant cerebral small vessel disease in the basal ganglia region including lacunae, white matter hyperintensity and perivascular spaces, *n* = 5; (9) with brain lesions, *n* = 2; (10) with history of concussion, *n* = 1. After exclusion, 85 PD and 140 NC were included in the study.

For PD patients who were under antiparkinsonian treatment, clinical assessments and MRI scanning were performed in the morning after withdrawing all antiparkinsonian drugs overnight (at least 12 h) (on “drug-off status”). Basic demographic information, such as age, gender, education, and disease duration, and neurologic and psychiatric scales including Unified Parkinson’s Disease Rating Scale (UPDRS), Hoehn-Yahr stage, Mini-Mental State Examination (MMSE) score were obtained from all PD patients. For NC, basic demographic information, and MMSE score were recorded.

### MRI data acquisition

All participants were scanned on a 3.0-Tesla MRI scanner (GE Discovery 750) equipped with an 8-channel head coil. During MRI scanning, the head was stabilized using restraining foam pads, and earplugs were provided to reduce the noise during scanning. Enhanced susceptibility-weighted angiography (ESWAN) was acquired to generate QSM and quantify the nigral iron content, while structural T1-weighted image and rsfMRI images were acquired to measure brain function. Structural T1 images were acquired using a fast-spoiled gradient recalled sequence: repetition time = 7.336 ms; echo time = 3.036 ms; inversion time = 450 ms; flip angle = 11°; field of view = 260×260 mm^2^; matrix = 256×256; slice thickness = 1.2 mm; 196 continuous sagittal slices. Enhanced susceptibility-weighted angiography images were acquired using gradient recalled echo sequence: repetition time = 33.7 ms; first echo time/spacing/eighth echo time = 4.556 ms/3.648 ms/ 30.092 ms; flip angle = 20°; field of view = 240×240 mm^2^; matrix = 416×384; slice thickness = 2 mm; slice gap = 0 mm; 64 continuous axial slices. The rsfMRI images were acquired using gradient recalled echo-echo planar imaging sequence: repetition time = 2000 ms; echo time = 30 ms; flip angle = 77°; field of view = 240×240 mm^2^; matrix = 64×64; slice thickness = 4 mm; slice gap = 0 mm; 38 interleaved axial slices.

### QSM data processing and semi-automatic segmentation of SN in QSM

**Fig. 1:**
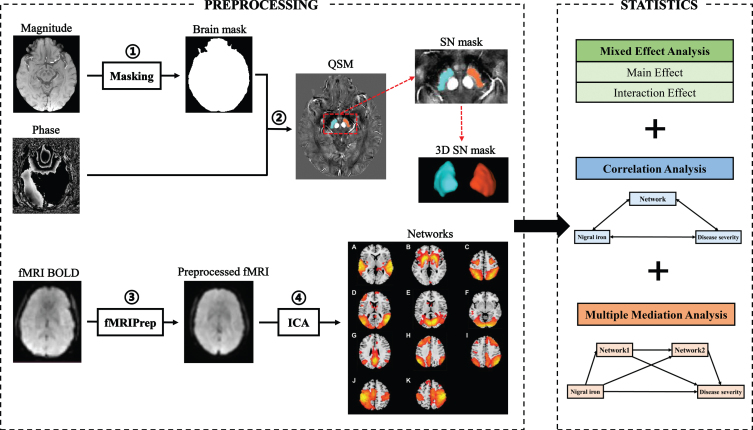
Flow chart of data processing and analysis. Magnitude and phase images are acquired with GRE sequence. The magnitude image is used to create a brain mask (①). The QSM image was generated by using phase image and the brain mask (②). The rsfMRI processing is performed using fMRIPrep (③). All preprocessed rsfMRI data are analyzed using ICA (④). Smith’s template was used to identify 11 brain networks from 49 estimated components, including auditory network (A), basal ganglia network (B), dorsal attention network (C), lateral visual network (D), medial visual network (E), occipital visual network (F), default mode network (G), left frontoparietal network (H), right frontoparietal network (I), left sensorimotor network (J), and right sensorimotor network (K). Statistical methods include mixed effect analysis, correlation analysis, and multiple mediation analysis. GRE, gradient echo; QSM, quantitative susceptibility mapping; rsMRI, resting-state magnetic resonance imaging; ICA, independent component analysis.

Susceptibility Tensor Imaging Suite V3.0 software package (https://people.eecs.berkeley.edu/∼chunlei.liu/software.html) was used to calculate the susceptibility maps from the phase images. Specifically, the raw phase was unwrapped using a Laplacian-based phase unwrapping [15], and the normalized phase was calculated. The normalized background phase was removed using the spherical-mean-value filtering (V_SHARP) [16]. QSM images were calculated using STAR-QSM (STreaking Artifact Reduction for QSM) method [17]. The mean signal from the individual brain was used as a susceptibility reference (see Fig. 1).

The tissue susceptibility of native SN was extracted by using a semi-automatic segmentation method on the ANTs-R language environment as shown in the previous study [18]: 1) by using ANTs-SyN coregistration algorithms [19], the native QSM image was registered to a newly constructed QSM template derived from a cohort of aging brains [20]; 2) the labels of bilateral SN were defined in the QSM template (Fig. 1); 3) the labels in the QSM template were then warped to the native QSM image space by inverting the transformation matrix calculated in the first step; 4) manual refinement was performed to ensure the segmentation precision by a neuroradiologist with 7 years of experience. Finally, the mean tissue susceptibility of bilateral SN was calculated, indicating the iron content in SN.

Since no available method is defined for identifying subjects with high nigral iron content, a median split way was suggested previously to identify subjects with high nigral iron content, which was constructed on the median nigral magnetic susceptibility of the NC group (0.095 ppm) [21]: susceptibility values above or equal to 0.095 ppm were categorized as “high SN magnetic susceptibility”, otherwise were identified as “low SN magnetic susceptibility”. In this way, we divided PD and NC into PD patients with high nigral iron (PD-SN_high_), PD patients with low nigral iron (PD-SN_low_), NC with high nigral iron (NC-SN_high_), and NC with low nigral iron (NC-SN_low_).

In order to verify the rationality of this grouping method, we used SN iron as a continuous variable to verify the results of interaction analysis in SPSS (see Supplementary Table 1 for details).

### rsfMRI preprocessing and ICA analysis

The rsfMRI processing was performed using fMRIPrep v20.2.1 (https://fmriprep.org/en/20.2.1/) [22] with the default processing steps (Fig. 1). To summarize: each T1-weight image was corrected for intensity non-uniformity and skull-stripped. Brain surfaces were reconstructed using recon-all from FreeSurfer software. Spatial normalization to the ICBM 152 Nonlinear Asymmetrical template version 2009c was performed through nonlinear registration, using brain-extracted versions of both the T1-weighted images and template. Brain tissue segmentation of cerebrospinal fluid, white matter, and gray matter was performed on the brain-extracted T1-weight images. Functional data were corrected for slice-timing, motion, and field distortion. This was followed by co-registration to the corresponding T1-weighted images using boundary-based registration with 9 degrees of freedom. All processed rsfMRI data were denoised by fMRI Denoise (https://github.com/compneuro-ncu/fmridenoise) with the default procedures, including temporal band-pass filtering (0.008-0.08 Hz), detrending, and regression of the nuisance covariates (24 head motion parameters, white matter, and cerebrospinal fluid confound, and framewise displacement). Finally, all functional data were resampled to 3 mm isotropic and smoothed with a 5 mm full width at half maxima Gaussian kernel, masked by gray matter.

All preprocessed rsfMRI data of PD patients and NC were analyzed using a Group ICA toolbox (Group ICAT version 4.0b). Independent components (IC) estimation mainly included three steps: data reduction, application of the ICA algorithm, and back-reconstruction. The data dimensionality was reduced using two steps of principal component analysis, and the optimal number of IC was estimated using the minimum description length algorithm (49 components were finally estimated). Then, the Informix algorithm was used to run the ICA [23]. Finally, the temporospatial back-reconstruction method was used to generate time courses and spatial maps for each participant.

Finally, we used Smith’s template to extract 11 brain networks from 49 estimated components [24] (Fig. 1A-K).

### Statistical analyses

#### Demographic and clinical data analyses

Tests for differences in demographic, clinical, neuropsychological, or imaging-based parameters between subjects in the NC and PD group were performed using the IBM SPSS 26.0 statistical software for Windows. Regarding the demographics and clinical data analyses, the Chi-square test was used for gender distribution difference assessment (*p* < 0.05 was regarded as significant). Analysis of variance (ANOVA) was used to compare the education and age among four groups. Post hoc analysis of two-sample t-test was performed afterward between the two subtypes (PD high iron vs. PD low iron, NC high iron vs. NC low iron) (Tukey’s test corrected, *p* < 0.05 was regarded as significance). Then, general linear model was used to compare the UPDRS score, MMSE score, Hoehn-Yahr stage, and iron content in SN with regressing out gender, age, and education (Tukey’s test corrected, *p* < 0.05 was regarded as significance). Disease duration in two PD groups was compared by independent samples t-test.

#### Imaging analyses

The statistical analyses of imaging data were conducted and visualized using the DPABI toolbox [25]. We extracted 11 functional networks from 49 independent components estimated by ICA in all groups. One sample t-test was performed to identify the overview of these functional networks (*q* < 0.01, false discovery rate (FDR) corrected). Specifically, we performed a 2×2 mixed effect analysis to explore the main effect of PD status and nigral iron, respectively. Meanwhile, the potential interaction effect between PD status and nigral iron in these networks was explored. In these functional analyses, age, gender, and education were regressed out as covariates of no interest, and the multiple-comparison correction was conducted by using the Gaussian Random Field (GRF) method with voxel *p* < 0.005, cluster *p* < 0.05 [26]. To further understand how PD status and nigral iron interacted on regional brain activities, we extracted mean network-based FC from ICA images within each cluster showing significant interaction effect and performed general linear model to detect the changes of these clusters among four groups with regressing out age, gender and education in IBM SPSS Statistics 26.0. To explore the clinical significance of imaging metrics, partial correlation analysis was conducted with adjusting the same covariates mentioned above.

It is worth noting that, since the FC alteration trend of brain clusters showing interaction effect in lVN and mVN was similar, we calculated the global FC of these clusters in VN as follows: 1) both FC in lVN and mVN were standardized by employing Z transformation; 2) the sum of each Z-transformed FC was computed as a global FC of clusters showing interaction effect in VN.

#### Multiple mediation analysis

Based on the demonstrated associations among the nigral iron content, the FC of brain networks, and UPDRS II score, as well as the further finding of the statistically significant total effect of nigral iron on UPDRS II score, we conducted multiple mediation analysis to test that whether the FC of brain networks was a mediator between nigral iron deposition and UPDRS II score in PD [27–30]. By permutations and combinations, we explored the mediation effects of all the interaction effect clusters obtained by the mixed effect analysis. In addition, for multiple interaction effect clusters in the same network, both individual and aggregate FC mediations were analyzed. A statistic toolbox (PROCESS Procedure for SPSS Release 2.16.3, http://www.afhayes.com/index.html) was used. The indirect effect of the FC of brain networks on nigral iron content and UPDRS II score was estimated by using bootstrapping approach with 5000 resampling [31]. To derive the 95% confidence interval, the elements of the vector of 5000 estimates of indirect effect were sorted from low to high. In the sorted distribution of these estimates, the lower limit of the confidence interval is defined as the 125^th^ estimate and the upper limit is defined as the 4875^th^ estimate. The outcome of indirect effect was considered as statistically significant (*p* < 0.05, two-tailed) when zero is not included in the 95% confidence interval [31].

## RESULTS

### Characteristics of the study population

Demographic, clinical, and imaging data were shown in Table 1. According to the median QSM of SN in NC (0.095 ppm), 140 NC and 85 PD were divided into four groups: NC-SN_high_ (*n* = 70), NC-SN_low_ (*n* = 70), PD-SN_high_ (*n* = 58), and PD-SN_low_ (*n* = 27). Thus, in both NC and PD groups, nigral iron content in the high iron subgroups was significantly higher than that in the low iron subgroups (*p* < 0.001). And iron content in SN in PD was significantly higher than that in NC (*p* < 0.001).

**Table 1:** Overview of sample demographics, clinical data as well as imaging parameters

	NC-SN_high_	NC-SN_low_	P_1_	PD-SN_high_	PD-SN_low_	P_2_	P_3_	P_4_
Demographic data
Number (n)	70	70	/	58	27	/	/	/
Age (y)	60.26±7.15	60.15±7.38	1.00	61.53±8.77	60.48±7.85	0.94	0.20	0.75
Education (y)	10.26±3.54	9.67±3.90	0.83	7.20±4.55	8.35±4.66	0.62	<0.001	<0.001
Gender (male/female)	33/37	35/35	0.74	29/29	12/15	0.63	0.96	0.95
Disease duration (y)	/	/	/	5.15±4.57	5.02±6.18	0.35	/	/
Hoehn-Yahr stage	/	/	/	2.44±0.38	2.35±0.43	0.37	/	/
Clinical data
MMSE score	27.83±1.89	28.26±1.78	0.39	25.22±5.47	27.30±2.61	0.10	0.007	0.002
UPDRS I score	/	/	/	1.29±1.46	1.44±1.53	0.63	/	/
UPDRS II score	/	/	/	10.31±4.73	7.74±4.52	0.02	/	/
UPDRS III score	/	/	/	27.28±11.11	22.70±13.49	0.19	/	/
Imaging parameters
SN QSM (ppm)	0.11±0.01	0.78±0.01	<0.001	0.12±0.02	0.08±0.01	<0.001	<0.001	<0.001

NC-SN_high_, NC with high nigral iron; NC-SN_low_, NC with low nigral iron; PD-SN_high_, PD patients with high nigral iron; PD-SN_low_, PD patients with low nigral iron; MMSE, Mini-Mental State Examination; UPDRS, Unified Parkinson’s Disease Rating Scale; SN, substantia nigra; QSM, Quantitative Susceptibility Mapping. Grouping is based on the median nigral iron content of NC (0.095 ppm). P_1_, differences between NC groups; P_2_, differences between PD groups; P_3_, differences between NC and PD groups; P_4_, differences among four groups. Values are expressed as mean±standard deviation.

No significant difference in age (*p* = 0.75) and gender (*p* = 0.95) was observed among four groups, while significant difference in education was observed (*p* < 0.001). Between PD groups, no significant difference in disease duration (*p* = 0.35) and Hoehn-Yahr stage (*p* = 0.37) was found. Further analysis showed that no significant difference was found in MMSE score between two NC groups (*p* = 0.39) and PD groups (*p* = 0.10). In addition, the UPDRS II score (*p* = 0.02) in PD-SN_high_ was significantly higher than that in PD-SN_low_, indicating that PD with higher nigral iron would have poor clinical profiles. However, no significant difference was found in UPDRS I score (*p* = 0.63) and UPDRS III score (*p* = 0.19) between the two PD groups.

### Identification of resting-state brain networks by ICA

Eleven resting-state networks were extracted and shown in Fig. 1A-K, including auditory network (AN), BGN, dorsal attention network (DAN), lateral visual network (lVN), medial visual network (mVN), occipital visual network (oVN), default mode network (DMN), left frontoparietal network (lFPN), right frontoparietal network (rFPN), left sensorimotor network (lSMN), and right sensorimotor network (rSMN) (FDR *q* < 0.01).

### Mixed effect analysis: main effect and PD*iron interaction effect on network function

**Fig. 2:**
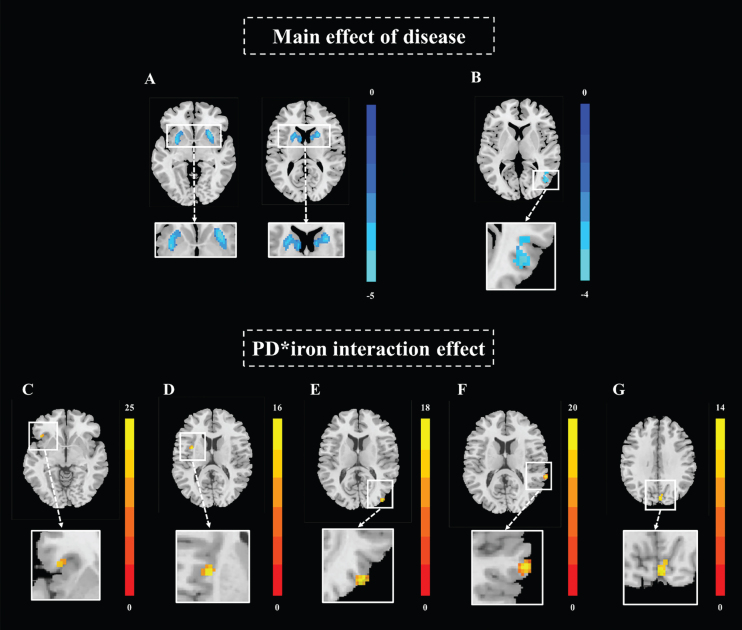
Main effect of disease, and interaction effect between nigral iron and disease on BGN and VN. (A) Main effect of disease on BGN; (B) main effect of disease on VN; (C) interaction effect cluster1 in BGN; (D) interaction effect cluster2 in BGN; (E) interaction effect cluster1 in lVN; (F) interaction effect cluster2 in lVN; (G) interaction effect cluster in mVN. All results are displayed at GRF correction (voxel level *p* < 0.005, cluster level *p* < 0.05). Color bars are proportional to T/F values. BGN, basal ganglia network; VN, visual network; lVN, lateral visual network; mVN, medial visual network; GRF, Gaussian Random Field.

Based on voxel-based mixed effect analysis, the main effect of disease was observed in these networks: 1) BGN: left caudate and putamen, as well as right caudate and putamen (peak MNI coordinate: *X* = –27.5/28.5, *Y* = 13.5/11.5, *Z* = –8.5/-8.5; peak *T* = –5.4/-5.9, respectively) (Fig. 2A); 2) VN: right middle temporal and occipital gyrus (peak MNI coordinate: *X* = 44.5, *Y* = –70.5, *Z* = 9.5; peak *T* = –4.4) (Fig. 2B). Notably, no nigral iron main effect brain cluster was found, indicating that nigral iron deposition alone did not have significant effect on the brain functional network.

Significant interaction effect between PD status and nigral iron were observed in the following networks: 1) BGN: left inferior frontal gyrus and left insular lobe (peak MNI coordinate: *X* = –41.5/-33.5, *Y* = 21.5/3.5, *Z* = –2.5/13.5; peak *F* = 15.8/16.5, respectively) (Fig. 2C, D); 2) VN: right middle occipital gyrus, right superior temporal gyrus, and bilateral cuneus (peak MNI coordinate: *X* = 42.5/66.5/4.5, *Y* = –86.5/-38.5/-82.5, *Z* = 11.5/13.5/33.5; peak *F* = 18.6/22.0/14.4, respectively) (Fig. 2E-G) (Table 2).

**Table 2:** Mixed effect analysis results in resting-state networks

Brain cluster	Main brain region	Peak MNI coordinate			Peak intensity (T/F value)	Cluster size
		X	Y	Z		
Main effect of disease
Cluster1 in BGN	Putamen_L, Caudate_L	–27.5	13.5	–8.5	–5.4^‡^	562
Cluster2 in BGN	Putamen_R, Caudate_R	28.5	11.5	–8.5	–5.9^‡^	625
Cluster in lVN	Temporal_Mid_R, Occipital_Mid_R	44.5	–70.5	9.5	–4.4^‡^	116
Interaction effect
Cluster1 in BGN	Frontal_Inf_L	–41.5	21.5	–2.5	15.8^†^	21
Cluster2 in BGN	Insula_L	–33.5	3.5	13.5	16.5^†^	25
Cluster1 in lVN	Occipital_Mid_R	42.5	–86.5	11.5	18.6^†^	29
Cluster2 in lVN	Temporal_Sup_R	66.5	–38.5	13.5	22.0^†^	48
Cluster in mVN	Cuneus_R, Cuneus_L	4.5	–82.5	33.5	14.4^†^	32

Peak intensity: ^‡^T value, ^†^F value; MNI, Montreal Neurological Institute; BGN, basal ganglia network; lVN, lateral visual network; mVN, medial visual network.

**Fig. 3:**
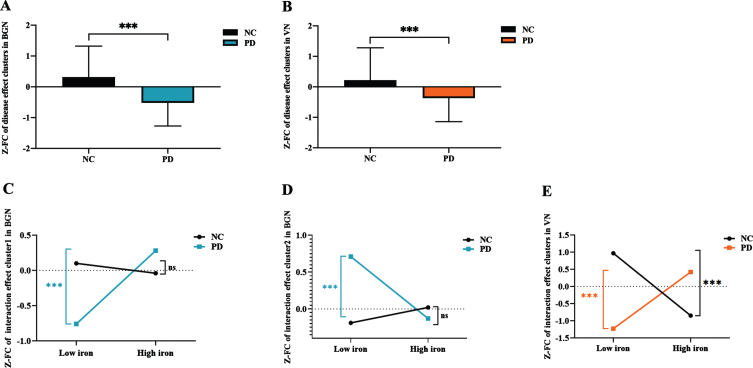
The FC alterations in the networks showing significant main effect of disease and disease*iron interaction effect. (A) The FC of disease main effect clusters in BGN; (B) the FC of disease main effect cluster in VN; (C) the FC of interaction effect cluster1 in BGN; (D) the FC of interaction effect cluster2 in BGN; (E) the FC of interaction effect clusters in VN. ns: *p* > 0.05; ***p≤0.001. In BGN, PD is depicted in blue and NC in black; In VN, PD is depicted in orange and NC in black. FC, functional connectivity; BGN, basal ganglia network; VN, visual network; PD, Parkinson’s disease; NC, normal control; Z, Z transformation.

In the post-hoc analysis, we found that the FC of brain clusters with significant main effect of disease in both BGN (Fig. 3A) and VN (Fig. 3B) was significantly decreased in PD compared with NC (both *p* < 0.001).

As for the brain clusters showing significant interaction effect between PD status and nigral iron, the FC of left inferior frontal gyrus in BGN was significantly higher in PD-SN_high_ than that in PD-SN_low_ (*p* = 0.001), while the FC was not significantly different between NC-SN_high_ and NC-SN_low_ (*p* = 0.396) (Fig. 3C). And the FC of left insular lobe in BGN was significantly lower in PD-SN_high_ than that in PD-SN_low_ (*p* = 0.001), while the FC of these brain clusters was not significantly different between NC-SN_high_ and NC-SN_low_ (*p* = 0.227) (Fig. 3D). Moreover, we observed that the FC of these clusters in VN remained significantly increased in PD-SN_high_ compared with PD-SN_low_ (*p* = 0.001), while that was decreased in NC-SN_high_ compared with NC-SN_low_ (*p* < 0.001) (Fig. 3E).

### Correlations among nigral iron content, the FC of brain clusters with interaction effect, and UPDRS II score

**Fig. 4:**
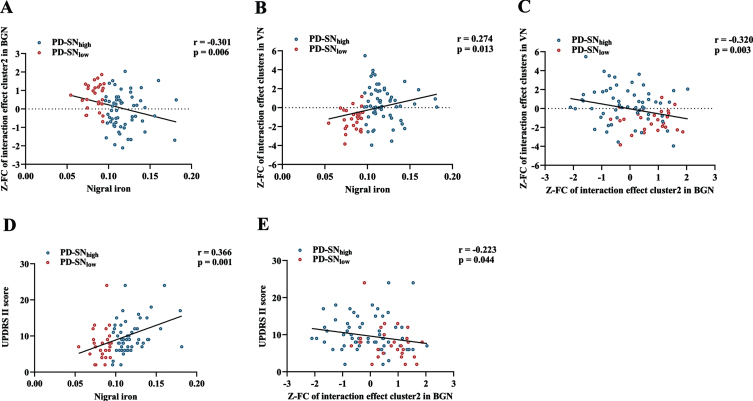
Correlations among nigral iron content, the FC of interaction effect clusters, and UPDRS II score in PD. (A) The correlation between nigral iron content and the FC of interaction effect cluster2 (left insular lobe) in BGN. (B) The correlation between nigral iron content and the FC of interaction effect clusters in VN. (C) The correlation between the FC of interaction effect cluster2 (left insular lobe) in BGN and the FC of interaction effect clusters in VN. (D) The correlation between nigral iron content and UPDRS II score. (E) The correlation between the FC of interaction effect cluster2 (left insular lobe) in BGN and UPDRS II score. *p* < 0.05 indicates significant correlation. PD-SN_high_ is depicted in blue and PD-SN_low_ is in red. FC, functional connectivity; UPDRS, Unified Parkinson’s Disease Rating Scale; PD, Parkinson’s disease; BGN, basal ganglia network; VN, visual network; PD-SN_high_, PD patients with high nigral iron; PD-SN_low_, PD patients with low nigral iron; Z: Z transformation.

Through partial correlation analysis (controlling for gender, age, and education), nigral iron content was significantly positively correlated with UPDRS II score in PD (*r* = 0.366; *p* = 0.001) (Fig. 4D). For BGN, nigral iron content was significantly negatively correlated with the FC of left insular lobe (*r*=-0.301; *p* = 0.006) (Fig. 4A), which was negatively correlated with UPDRS II score (*r*=-0.223; *p* = 0.044) (Fig. 4E). For VN, nigral iron content was significantly positively correlated with the FC of right middle occipital gyrus, right superior temporal gyrus, and bilateral cuneus (*r* = 0.274; *p* = 0.013) (Fig. 4B). Furthermore, there was a significant negative correlation between the FC of brain clusters showing interaction effect in BGN and VN in PD (*r*=-0.320; *p* = 0.003) (Fig. 4 C).

### Multiple mediation effect of the FC of clusters with interaction effect on the relationship between nigral iron content and UPDRS II score

**Fig. 5:**
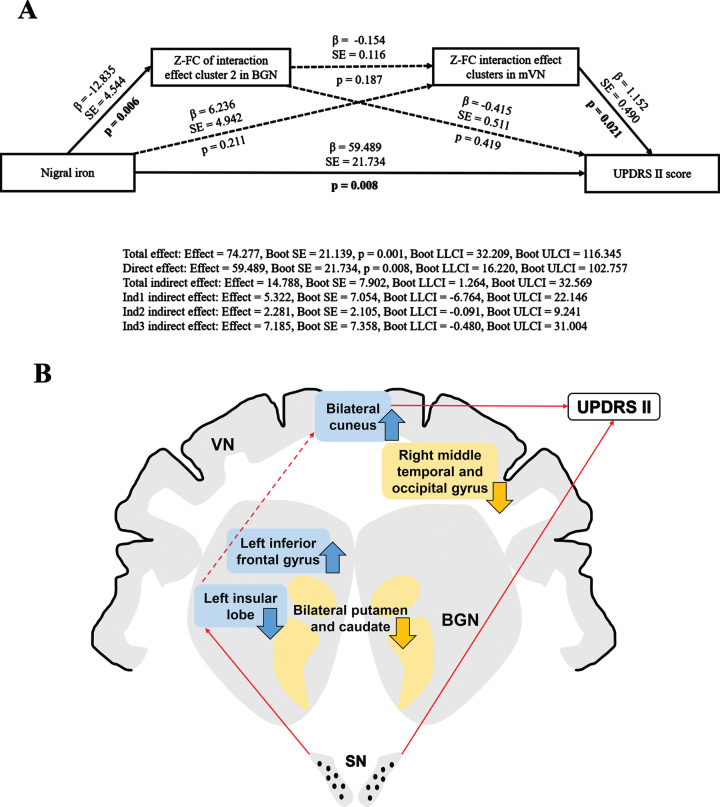
(A) Multiple mediation effects of the FC on the relationship between nigral iron deposition and UPDRS II score in PD. Paths that are statistically significant are displayed with standardized coefficients and standard error after bootstrapping on solid lines, whereas paths that are not statistically significant are presented as dashed lines. Ind1 indirect effect: indirect effect of the FC of interaction effect cluster2 (left insular lobe) in BGN; Ind2 indirect effect: indirect effect of the FC of interaction effect cluster in mVN; Ind3 indirect effect: chain mediation effect of the FC of interaction effect clusters in BGN and mVN. (B) Full view of the mediation analysis results. The FC of interaction effect clusters in both BGN and mVN partially mediate the relationship between nigral iron and UPDRS II score, but they do not perform as single or chained mediators. The blue rectangle represents interaction effect clusters in BGN and mVN. The yellow rectangle represents the main effect of disease clusters in BGN and VN. The black dots in the SN represent iron deposition. The upward arrow represents FC elevation; The downward arrow indicates FC descent. The red solid line represents *p* < 0.05; The red dashed line indicates *p* > 0.05. β, standardized coefficients; Boot SE, bootstrap standard error; Boot LLCI, bootstrap lower limited confidence interval; Boot ULCI, bootstrap upper limited confidence interval; Z, Z transformation; FC, functional connectivity; UPDRS, Unified Parkinson’s Disease Rating Scale; BGN, basal ganglia network; mVN, medial visual network; PD, Parkinson’s disease. VN, visual network; SN, substantia nigra.

Multiple mediation analysis revealed that the FC of interaction effect clusters in both BGN and mVN may partially mediate the association between nigral iron content and UPDRS II score (Total indirect effect: Effect = 14.788, Boot SE = 7.902, Boot LLCI = 1.264, Boot ULCI = 32.569). In other words, under separate conditions, neither the FC of interaction effect brain clusters in BGN nor mVN could mediate the relationship between nigral iron and UPDRS II score (Ind1 indirect effect: Effect = 5.322, Boot SE = 7.054, Boot LLCI = –6.764, Boot ULCI = 22.146; Ind2 indirect effect: Effect = 2.281, Boot SE = 2.105, Boot LLCI = –0.091, Boot ULCI = 9.241, respectively). And the direct effect was statistically significant (Direct effect: Effect = 59.489, Boot SE = 21.734, *p* = 0.008, Boot LLCI = 16.220, Boot ULCI = 102.757), which indicated that nigral iron deposition might be an effector of disease to worsen. Moreover, the FC of interaction effect clusters in BGN and mVN did not serve as chain mediators for the relationship between nigral iron and UPDRS II score (Ind3 indirect effect: Effect = 7.185, Boot SE = 7.358, Boot LLCI = –0.480, Boot ULCI = 31.004). This suggested that the FC of BGN and mVN might play an important role in mediating nigral iron deposition and disease severity, and neither was indispensable. Since there was no chain mediation effect between BGN and mVN, there might be other mediators between them (Fig. 5).

## DISCUSSION

In this study, we decoded the respective main effect of PD status and nigral iron deposition and their potential interaction effect on brain functional networks using multi-modality MRI technology, further explored their relationship to disease severity. The main findings were as follows: First, the main effect of disease was observed in bilateral caudate and putamen in BGN, as well as right middle temporal and occipital gyrus in VN, both of which showed significantly decreased FC in PD compared with NC. Notably, we did not find the main effect of nigral iron on brain functional networks. Further analysis revealed that brain clusters showing significant interaction effect between PD status and nigral iron was the left inferior frontal gyrus and left insular gyrus in BGN, as well as right middle occipital gyrus, right superior temporal gyrus, and bilateral cuneus in VN. In detail, for BGN, PD-SN_high_ showed significantly increased FC in inferior frontal gyrus and decreased FC in left insular lobe compared with PD-SN_low_; for VN, PD-SN_high_ had significantly higher FC than PD-SN_low_, while NC-SN_high_ had significantly decreased FC compared with NC-SN_low_. Finally, based on the significant correlations between nigral iron, the FC of clusters showing interaction effect in BGN and VN, and UPDRS II score observed in PD patients, further multiple mediation analysis was performed and revealed that the FC of these clusters in both BGN and mVN may partially mediate the relationship between nigral iron content and UPDRS II score.

For the main effect of PD, significantly reduced FC in the bilateral caudate and putamen of BGN was observed, while its interaction effect with nigral iron was exclusively detected in BGN, where increased FC in the inferior frontal gyrus and reduced FC in the insular lobe were observed in PD-SN_high_ compared with PD-SN_low_. The nigral iron deposition has been proved to be one of the important pathologic changes in PD [3]. BGN has direct dopaminergic connectivity from SN [32] and widespread connectivity to multiple specific brain regions [8, 33], thus its dysfunction resulting from nigral degeneration might play a core role in PD pathophysiology. Consistent with this finding, several fMRI studies have demonstrated functional abnormalities in the putamen and caudate nucleus of basal ganglia in PD [34, 35] as well as the dysfunction of BGN [36]. Further, a potential pathway linking iron-related nigral degeneration to global disruption of weighted functional topology mediated by striatal dysfunction was reported in PD [33]. However, these previous studies mixed the effects of PD status and nigral iron on brain function and did not take their interaction into account, which has been acknowledged by histopathology [5]. This study separated these two effects and discovered the interaction effect of PD status and nigral iron deposition on BGN function. For the clusters with interaction effect in BGN, insula has been found with the involvement of a-synuclein deposition, associated with a disruption of normal neurotransmitter function, connectivity alteration, and metabolic and structural changes in PD [37]. And based on the dense connectivity between insular and frontal regions [38], our findings of decreased FC in insula and increased FC in frontal regions further indicated that these brain areas might have the intrinsic ability to jointly keep a relative balance of function when nigral iron and PD status simultaneously or sequentially occurred. Therefore, current findings suggested that striatum, which occupied the core position in BGN, was specifically disconnected in PD; and, by combing the negative finding in NC, our results suggested that single nigral iron deposition may not be sufficient enough to interrupt FC change in BGN in NC, only when the PD status participated, BGN function was significantly perturbed.

Another damaged network identified in PD was VN. The main effect of disease was located in the right middle temporal and occipital gyrus of VN, demonstrating reduced FC under PD status, but with little effect of nigral iron. Many studies were exploring the changes of vision-related brain regions in PD. Consistent with our results, brain network studies revealed decreased temporal-occipital connectivity in PD, which strongly suggested the involvement of the visual cortex in PD [39]. Then, in the brain regions showing interaction effect between PD status and nigral iron, PD-SN_high_ had significantly higher FC than PD-SN_low_ in VN, while NC-SN_high_ had significantly decreased FC compared with NC-SN_low_. Dopamine homeostasis is very important in maintaining normal brain function and its high-efficiency performance, and it is worth noting that VN is partially innervated by dopamine though not so many as BGN [40]. The excessive iron deposition in SN would lead to high oxidative stress on dopaminergic neurons more or less [41], therefore, mild nigral degeneration might occur when iron-related oxidative stress exceeds its protective capacity in aging, which may explain the decreased FC within VN in NC. And sufficient evidence has demonstrated that under PD status, the nigral iron accumulation would accelerate the nigral degeneration and associated with the plummet of brain dopamine [42], thus the significant disruption of BGN and VN are expected. As well documented, the activation of VN in PD patients may be a compensation for dyskinesia [43–46]; therefore, nigral neurodegeneration would have an effect on VN, which was not well studied, neither its relationship with nigral iron deposition in PD. In the current data-driven results, we observed that nigral iron deposition was closely associated with FC decline in BGN (Fig. 3D) and FC enhancement in VN (Fig. 3E), indicating that visual function could keep a critical role in reserving BGN function when nigral iron overload occurred in PD. Moreover, the finding of multiple mediation analysis further strengthened and demonstrated previous findings. Taken together, these findings suggested that in normal aging, high nigral iron would negatively affect VN function, while its overactivity would arise to reserve BGN function which is the core target to dopaminergic depletion resulting from the irresistible iron-related nigral degeneration under PD status.

Moreover, as mentioned in the results, significant relationships among nigral iron, BGN, VN, and disease severity (i.e., UPDRS II) were detected, and further mediation analysis disclosed that the FC of both BGN and mVN might play a partial mediation role for the nigral iron to effect disease severity in PD patients, suggesting that nigral iron deposition could not only directly influence disease severity but also affect disease severity through FC changes of BGN and mVN. Previous studies have well demonstrated that dopamine depletion is both associated with motor and non-motor symptoms [47–49]. Therefore, UPDRS II score has been suggested to be a better marker of disease progression than the UPDRS III score, that was taking non-motor symptoms into consideration [50, 51]. As far as we know, how the iron-related nigral degeneration influenced PD behavior remains largely unknown, and this network-based iron-related neural circuit gave a new insight into the PD status by employing multi-modality MRI, which was summarized in the Fig. 5B and exploring a potential causal mediation pathway of nigral iron deposition to influence disease severity that was mediated by both BGN dysfunction and VN hyperfunction in PD. However, here we did not detect significant chain mediator effect of BGN and mVN, which might be limited by the numerous unknown factors within and beyond this complicated neural circuit. Therefore, future studies are warranted to extend our findings and display the whole picture of PD-related neural circuits as comprehensively as possible.

We speculated the reasons of lacking correlation between SN iron content and UPDRS III as follows: Even though the included PD patients withdrew drugs for more than 12 h, UPDRS III remains easily influenced by dopaminergic therapy (especially LDR) varying from individual to individual [52]; therefore, in a number of published documents, the relationship between SN and UPDRS III has a high variation [53–59], and many of them did not find this significant relationship [54–58]. Thus, whether SN iron would specifically influence UPDRS III in PD is still an open issue, further studies, e.g., drug-naïve population, and longitudinal observation, may further clarify that.

### Limitations

First, currently, the diagnosis of PD mainly relies on the clinical symptoms, and no exact PD pathology of α-synuclein was acquired, which may bring certain unknown biases. Second, because nigral iron accumulation has been well established as an imaging biomarker for PD, the sample size of PD patients with low nigral iron was relatively small. Finally, patients with PD who received long-term drug therapy might experience reorganization and compensatory changes in brain function. Since more than two-thirds of the patients in this study were taking medication, the medical effects on brain function could not be avoided, even though all the patients having anti-parkinsonian drugs were terminated drug for more than 12 h in the present study.

### Conclusion

Our study demonstrates an interaction of nigral iron deposition and PD status on brain networks, that is, nigral iron deposition is associated with the change of brain network configuration exclusively when in PD. We identified a potential causal mediation pathway— for iron to affect disease severity that was mediated by both BGN dysfunction and VN hyperfunction in PD. All these findings provide neuroimaging evidence for better understanding PD pathogenesis from iron-related nigral degeneration to PD clinical disability.

## Supplementary Material

Supplementary Material
