# Supplementary material for: Nigral Iron Deposition Influences Disease Severity by Modulating the Effect of Parkinson’s Disease on Brain Networks
Source: J Parkinsons Dis. 2022 Dec 16;12(8):2479–92. doi: 10.3233/JPD-223372 (PMC9837680; doi:10.3233/JPD-223372)
Supplement: Supplementary Material [file jpd-12-jpd223372-s001.pdf]

Supplementary Material

Nigral Iron Deposition Influences Disease Severity by Modulating the Effect of Parkinson's Disease on Brain Networks

Supplementary Table 1. The PD status*SN iron (continuous) interaction effect and main effect on brain networks.

F (p value)	lVN interaction effect cluster1	lVN interaction effect cluster2	mVN interaction effect cluster	BGN interaction effect cluster1	BGN interaction effect cluster2
PD status * SN iron (*continuous*)	13.22 **(<0.001*)**	11.91 **(0.001*)**	9.14 **(0.003*)**	10.12 **(0.002*)**	3.21 (0.075)
PD status	10.59 **(0.001*)**	10.73 **(0.001*)**	10.75 **(0.001*)**	11.19 **(0.001*)**	5.46 **(0.02*)**
SN iron (*continuous*)	0.88 (0.35)	2.25 (0.14)	0.65 (0.42)	2.00 (0.16)	3.40 (0.07)

The values were shown as F (p value). *p<0.05

BGN, basal ganglia network; lVN, lateral visual network; mVN, medial visual network; PD, Parkinson's disease; SN, substantia nigra.
